# Prevalence and Clinical Features of Polyendocrine Metabolic Ovarian Syndrome in the Gulf Cooperation Council Countries: A Systematic Review and Meta-Analysis

**DOI:** 10.3390/healthcare14131826

**Published:** 2026-06-23

**Authors:** Lama Ali Buhran, Meshal Bader Almutairi, Shehata Farag Shehata, Syed Esam Mahmood, Awad Alsamghan, Ramy Mohamed Ghazy

**Affiliations:** 1 College of Medicine, King Khalid University, Abha 62529, Saudi Arabia; lama.buhran@gmail.com; 2 College of Medicine, King Saud bin Abdulaziz University for Health Sciences, Riyadh 1148, Saudi Arabia; 3 Department of Family and Community Medicine, College of Medicine, King Khalid University, Abha 62529, Saudi Arabia; 4 Tropical Health Department, High Institute of Public Health, Alexandria University, Alexandria 21561, Egypt

**Keywords:** polycystic ovary syndrome, PCOS, prevalence, GCC, Gulf countries, systematic review, meta-analysis, symptoms

## Abstract

**Background:** Polyendocrine metabolic ovarian syndrome (PMOS/PCOS) is the most common hormonal disorder in women of reproductive age and is linked to infertility as well as long-term metabolic and psychological problems. In the Gulf Cooperation Council (GCC) region, rising obesity, dietary changes, and sedentary lifestyles may be increasing its burden. However, prevalence estimates remain highly inconsistent due to differences in diagnostic criteria and measurement methods rather than true variation in disease rates. **Objective**: This study aimed to describe the situation by systematically pooling available evidence on the prevalence of PMOS among women in GCC countries and by summarizing the range of clinical features reported across included studies. **Methods:** We conducted a systematic review and meta-analysis following PRISMA 2020 guidelines. We searched five major bibliographic databases (PubMed, Scopus, Web of Science, Cochrane Library, and Embase) and the Google Scholar search engine for observational studies published up to 1 June 2026. Studies were eligible if they reported PMOS prevalence and related clinical features among women of reproductive age residing in GCC countries. After removing duplicates and screening 570 initially identified records, 25 studies met our inclusion criteria; 24 were included in the quantitative meta-analysis after excluding one high-risk study. Risk of bias was appraised using the Joanna Briggs Institute Checklist for Prevalence Studies. A random-effects meta-analysis using the DerSimonian-Laird method, combined with the Freeman-Tukey double arcsine transformation, was used to estimate the pooled prevalence. Heterogeneity was quantified using the I^2^ statistic and Cochran’s Q test. Subgroup analyses explored differences by country, diagnostic method, study setting, and publication period. Meta-regression was used to identify study-level factors that explained between-study variability. **Results:** Across 24 studies involving 77,890 women, the pooled prevalence of PMOS was 17.59% (95% CI: 12.98–23.40%). Country-level estimates ranged from 6.56% in Oman to 23.0% in Saudi Arabia. Heterogeneity across all analyses was extremely high (I^2^ = 99.6%), and meta-regression identified the diagnostic tool as the single most important source of variation, explaining 42.7% of between-study variance. Studies using structured clinical criteria (Rotterdam or NIH) yielded prevalence estimates around 13–14%, while those relying on self-report or physician diagnosis without standardized criteria reported considerably higher figures (20–37%). Common clinical features included menstrual irregularity (up to 100% of PMOS cases in clinical cohorts), hirsutism (5–100%), acne and oily skin (17–74%), and obesity (17–73%). Awareness of PMOS among women in the region was highly variable, ranging from under 3% to nearly 100%. **Conclusions:** PMOS is a significant public health concern across the GCC region. The markedly higher pooled prevalence combined with high rates of obesity and metabolic risk in this population calls for urgent, coordinated action. Standardizing diagnostic practices, investing in population-level screening, and developing culturally tailored awareness programs are essential steps toward reducing the clinical and social burden of PMOS.

## 1. Introduction

Polyendocrine metabolic ovarian syndrome (PMOS), formerly referred to as polycystic ovary syndrome (PCOS), is a complex, multifactorial endocrine condition that affects about 8–13% of women of reproductive age [1], with prevalence varying according to the population examined and the diagnostic criteria used [2]. In 2021, PMOS affected 65.8 million cases globally, with 1.18 million new cases and 0.58 million disability-adjusted life years (DALYs). Middle sociodemographic index regions experienced the fastest increases in prevalence, incidence, and DALYs. Adolescents aged 15–19 years had the highest incidence rates, while prevalence peaked among women aged 30–34 years [3]. It is characterized by a spectrum of reproductive, metabolic, and dermatologic manifestations including oligo/anovulation, hyperandrogenism, polycystic ovarian morphology, hirsutism, acne, and insulin resistance that contribute to substantial clinical and psychosocial challenges across the lifespan [4].

From clinical perspective, PMOS has far-reaching implications. It is the most common cause of ovulatory dysfunction, with oligo/anovulation contributing significantly to infertility risk [5]. In addition, it is increasingly associated with chronic conditions such as type 2 diabetes mellitus, dyslipidemia, and hypertension, as well as an elevated risk of endometrial cancer [6,7]. This growing burden has direct consequences on reproductive health, healthcare planning, and the management of chronic diseases. In addition to its biomedical consequences, PMOS has a significant impact on mental health and quality of life [8]. Affected women report higher rates of anxiety and depression [9], highlighting the need for comprehensive, multidisciplinary approaches to care.

Unfortunately, awareness of PMOS among women remains inadequate despite its increasing prevalence. Studies have reported significant gaps in knowledge regarding PMOS symptoms, risk factors, and potential complications [10]. Low awareness may contribute to delayed diagnosis and treatment, increasing the risk of reproductive and metabolic complications [11]. Furthermore, although the prevalence of PMOS-related symptoms appears to be rising, many women remain unaware of the condition and often do not seek medical care when symptoms occur [12]. These findings underscore the importance of improving PMOS awareness to facilitate early detection and management. In the Gulf Cooperation Council (GCC) nations which comprise Saudi Arabia, the United Arab Emirates (UAE), Oman, Qatar, Kuwait, and Bahrain PMOS has become a significant reproductive health issue. The burden of PMOS in the region may be influenced by the rising prevalence of obesity among women, which has been largely attributed to changing dietary patterns, reduced physical activity, and rapid urbanization [13,14,15]. Given the well-established association between obesity and PMOS, evidence indicates that excess body weight and obesity significantly contribute to worsening the signs and symptoms of PMOS [16]. Women in GCC countries appear to face a higher risk of developing PMOS, as indicated by the study on the burden of PMOS in the Middle East North Africa (MENA) region between 1990 and 2019 [17]. The combined prevalence of PMOS was found to be 30.0% (95% CI: 29.0–38.0%, I^2^ = 91.98%) [18].

Despite growing research interest, data on the epidemiological burden of PMOS in the GCC remains fragmented and methodologically inconsistent. Existing studies from GCC countries have reported highly variable prevalence rates, ranging from as low as 2% to as high as 56%, largely attributable to differences in diagnostic criteria, sampling strategies, and clinical cut-off points [18,19,20,21,22]. The meta-analysis by Alam et al. [18] focused exclusively on infertile women, a high-risk group in whom the reported PMOS prevalence of 30% cannot be extrapolated to the broader female population. Consequently, a substantial gap remains in understanding the actual burden of PMOS among all women in GCC countries. Furthermore, that review did not assess key clinical manifestations such as hirsutism, acne, obesity, or menstrual disturbances, nor did it report prevalence estimates for specific subpopulations, including adolescents or university students. Accordingly, there is a strong rationale for a new and more comprehensive systematic review to determine PMOS prevalence in all women beyond infertile cohorts across the GCC; describe the broader range of clinical features; and generate population-based evidence to inform public health screening strategies, guide resource distribution, and shape awareness initiatives, thereby directly complementing and expanding upon the earlier, more narrowly focused review.

## 2. Methods

### 2.1. Study Design and Protocol

This systematic review and meta-analysis were conducted in accordance with the Preferred Reporting Items for Systematic Reviews and Meta-Analyses (PRISMA) guidelines [23]. The study protocol was registered in Open Science Framework (OSF).

Eligibility Criteria

Inclusion Criteria:

- Study design: Primarily observational, with most employing cross-sectional designs.
- Geographic scope: Studies conducted in GCC countries.
- Population: Women of reproductive age (typically 15–45 years).
- Outcome measures: Studies reporting PMOS prevalence (or from which prevalence could be calculated) and describing associated symptoms.
- Diagnostic criteria: Any accepted definition, including Rotterdam criteria (≥2 of 3 features: oligo/anovulation, hyperandrogenism, or polycystic ovarian morphology), National Institute of Health (NIH) criteria (hyperandrogenism plus ovulatory dysfunction, after exclusion of other causes), or clinician/self-reported diagnosis.
- Publication period: Articles published in English or Arabic till 1 June 2026.

Exclusion Criteria:

Studies were excluded if they met any of the following conditions: (1) case reports, conference abstracts, editorials, letters to the editor, commentaries, or opinion articles; (2) narrative reviews, systematic reviews, or meta-analyses (although their reference lists were checked for relevant primary studies); (3) qualitative studies or interventional studies that did not report baseline PMOS prevalence; (4) animal studies or studies limited to male participants; (5) studies focusing on pregnant women or on women with specific comorbid conditions (e.g., diabetes, thyroid disease) without providing data for the general population; (6) duplicate reports based on the same study population; (7) studies included fewer than 100 participants; (8) studies only presented biochemical data without a definitive PMOS diagnosis; or (9) did not provide sufficient information for data extraction, even after attempts to contact the corresponding author.

### 2.2. Data Sources and Search Strategy

We searched five major bibliographic databases (PubMed, Scopus, Web of Science, Cochrane Library, and Embase) and the Google Scholar search engine for observational studies. The search strategy combined controlled vocabulary terms (e.g., MeSH terms) and free-text keywords related to the study topic (Table 1).

A manual review of the reference lists from all included studies and pertinent review articles was performed to detect any additional eligible studies that might have been overlooked in the electronic database search. Furthermore, key journals related to the subject area were hand-searched to maximize the completeness of the literature coverage. All potentially relevant citations identified through these manual methods were assessed using the same eligibility criteria as those applied in the primary search strategy. All identified citations were exported into EndNote X20 reference management software (Clarivate Analytics, Philadelphia, PA, USA), where duplicate records were systematically identified and removed. The deduplicated library was then exported to Microsoft Excel to facilitate the screening process.

#### Study Selection

Stage 1: Title and abstract screening

Two reviewers independently (L.A.B and M.B.A) examined the titles and abstracts of all records identified through the database searches. Each record was evaluated against an initial set of eligibility criteria, which considered the population. Records that were clearly inconsistent with these criteria were excluded. The reviewers were not blinded to the authors’ identities, journal titles, or publication years.

Stage 2: Full-text retrieval and assessment

The full texts of all articles that passed title and abstract screening, as well as those with uncertain eligibility, were retrieved. Two reviewers (L.A.B. and M.B.A.) independently assessed each full-text article against predefined inclusion and exclusion criteria. Reasons for exclusion at this stage were recorded for all ineligible studies. When full-text articles were not available through institutional access or interlibrary loan, the corresponding authors were contacted via email. Articles that remained unavailable after these attempts were excluded and documented as such in the PRISMA flow diagram.

Stage 3: Disagreement resolution

Disagreements between the two reviewers at either the title/abstract screening stage or the full-text assessment stage were resolved through a structured, sequential process. First, reviewers discussed each discrepancy by re-examining the eligibility criteria and, when necessary, the full text of the article. If consensus cannot be reached, a third reviewer (R.M.G) independently evaluated the study and made the final decision. For persistent disagreements involving multiple studies, a full-team consensus meeting was held to ensure agreement. Inter-rater reliability prior to resolution was high, with Cohen’s kappa coefficient indicating near-perfect agreement at both screening stages (κ = 0.83 for title/abstract screening and κ = 0.89 for full-text review).

### 2.3. Data Extraction

A standardized, pre-piloted data extraction form was developed in Microsoft Excel and tested on three randomly selected included studies prior to finalization. Two independent reviewers (L.A.B. and A.A.) extracted data from all eligible studies, including study characteristics (author, year, country, city, study design, setting, study period, sample size, and sampling method), participant demographics (age, marital status, educational level, employment status, nationality, and BMI categories), and PMOS-related outcomes. These outcomes included prevalence estimates (number of cases and prevalence with 95% confidence intervals), diagnostic criteria used (Rotterdam, NIH, Androgen Excess and Polycystic Ovary Syndrome Society (AE-PCOS Society), or self-reported clinician diagnosis), and diagnostic methods (clinical, biochemical, ultrasound, or questionnaire-based). In addition, clinical manifestations were extracted, including menstrual irregularities, hirsutism (with scoring system where reported), acne and oily skin, alopecia, acanthosis nigricans, obesity or overweight status, infertility (primary or secondary), metabolic comorbidities (such as diabetes, dyslipidemia, and hypertension), and other reported symptoms (e.g., fatigue, headache, dizziness, back pain, voice changes, abdominal pain, and difficulty losing weight). Disagreements between reviewers were resolved through discussion and consultation with senior author (R.M.G). For missing or unclear data, corresponding authors were contacted up. If data remained unavailable, the variable was recorded as “not reported” in the extraction form.

### 2.4. Risk of Bias Assessment

Risk of bias of the included studies was assessed using the Joanna Briggs Institute (JBI) Critical Appraisal Checklist for Prevalence Studies. Two independent reviewers (A.A. and M.B.A.) assessed each study against the nine-item checklist, which evaluates key domains including appropriate sampling frame, adequate sample size, complete description of study participants and setting, sufficient coverage of the target population, use of valid and reliable measurement methods, appropriate statistical analysis, adequate response rate, and management of non-responders. Each item was scored as “Yes” (1 point), “No” (0 points), “Unclear” (0 points), or “Not Applicable” (0 points), yielding a total score ranging from 0 to 9. Based on the total score, studies were categorized into three risk-of-bias levels: low risk (score 7–9), indicating high methodological quality with minimal bias; moderate risk (score 4–6), indicating some methodological concerns and potential for bias; and high risk (score 0–3), indicating major methodological flaws and a high likelihood of bias. Any disagreements between the two reviewers were resolved through discussion or consultation with a third reviewer (S.E.M/R.M.G). Studies rated as high risk of bias were excluded from the meta-analysis to ensure the robustness of the pooled prevalence estimate. The inter-rater agreement for the quality assessment was substantial (κ = 0.84).

### 2.5. Statistical Analysis

All statistical analyses were conducted using R software (version 4.3.2; R Foundation for Statistical Computing, Vienna, Austria). Publication bias was assessed through visual inspection of funnel plots and formally tested using Egger’s regression test and Begg’s rank correlation test, with *p* < 0.10 indicating potential asymmetry.

Given the expected clinical and methodological heterogeneity across included studies such as differences in diagnostic criteria, study populations, settings, and sample sizes a random-effects meta-analysis using the DerSimonian and Laird method was applied to estimate the pooled prevalence of PMOS with corresponding 95% confidence intervals (CIs). To stabilize variance, the Freeman–Tukey double arcsine transformation was used, particularly to address studies reporting very low or very high prevalence values and to ensure that pooled estimates and confidence intervals remained within plausible bounds (0–1).

Between-study heterogeneity was assessed using the I^2^ statistic, with values of 25–49%, 50–74%, and ≥75% indicating low, moderate, and substantial heterogeneity, respectively. In addition, Cochran’s Q test was reported, with *p* < 0.10 considered indicative of statistically significant heterogeneity.

Sensitivity analyses included leave-one-out meta-analysis to evaluate the influence of individual studies, as well as comparison of random-effects versus fixed-effects models to test the robustness of findings.

Results were visually presented using forest plots, where individual study estimates were displayed as squares proportional to their weights and the pooled estimate as a diamond representing the overall prevalence and its 95% CI. Subgroup analyses were performed to explore potential sources of heterogeneity based on GCC country, diagnostic criteria (Rotterdam, NIH, AE-PCOS Society, or self-reported diagnosis), publication year (before or after 2020), and study setting (clinical [hospital], community [general population], or academic [university/medical students]).

Furthermore, meta-regression analysis was conducted to explore sources of heterogeneity in PMOS prevalence estimates. Meta-regression extends the random-effects meta-analysis model by regressing study-level covariates that may explain between-study heterogeneity. The meta-regression model was expressed as θi^ = θ + β_1_X_1i_ + β_2_X_2i_ + … + β_k_X_ki_ + ε_i_ + ζ_i_, where, where θ1^ is the observed logit-transformed prevalence for study i, θ is the overall pooled effect, β_1_ to β_k_ are regression coefficients for study-level covariates X_1_ to X_k_, ε_i_ is the sampling error within study i, and ζ_i_ is the between-study random effect assumed to follow a normal distribution with mean zero and variance τ^2^. The following study-level covariates were examined as potential moderators of heterogeneity: assessment tool (categorized as Rotterdam/NIH criteria, self-report, physician diagnosis, ultrasound/clinical assessment, or ICD-10 codes), country (Saudi Arabia, UAE, Kuwait, Qatar, or Oman), and study setting (clinical [hospital], community [general population], or academic [university/medical students]).

Given the limited number of studies (k = 24), we employed a parsimonious approach to avoid model overfitting [24]. Separate univariable meta-regression models were fitted for each covariate, followed by a multivariable model combining significant predictors, adhering to the recommendation of including no more than one predictor per ten studies to maintain statistical power [25]. All meta-regression models used restricted maximum likelihood (REML) estimation for the between-study variance (τ^2^), and the Knapp-Hartung adjustment was applied to improve the accuracy of confidence intervals and hypothesis tests [26]. Model fit was assessed using the Akaike Information Criterion (AIC) and Bayesian Information Criterion (BIC), where lower values indicate better fit. The proportion of between-study variance explained by each model was calculated as R^2^. The QM test was used to test the null hypothesis that all regression coefficients (except the intercept) are zero, with a significant QM statistic (*p* < 0.05) indicating that the covariate(s) significantly explain heterogeneity. The degree of heterogeneity not explained by the models was quantified using the I^2^ statistic, representing the percentage of total variation across studies attributable to heterogeneity rather than chance [27].

## 3. Results

**Figure 1:**
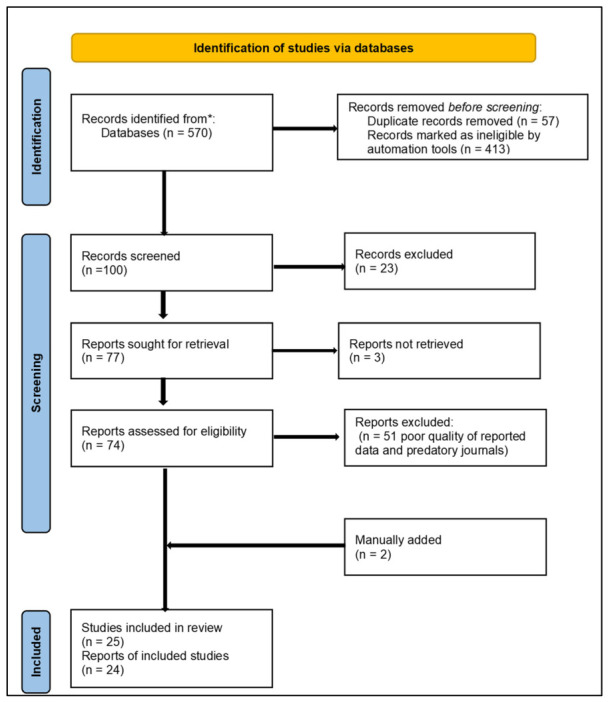
PRISMA flow diagram of study selection and inclusion. * the number of records identified from each database or register.

A total of 570 records were identified through database searching. Before screening, 57 duplicate records were removed, and an additional 413 records were excluded by automated screening tools, leaving 100 records for title and abstract screening. Of these, 23 records were excluded based on the title and abstract review. The remaining 77 reports were sought for retrieval; however, 3 reports could not be obtained. Consequently, 74 full-text articles were assessed for eligibility. At the full-text review stage, 51 articles were excluded because they were either published in predatory journals or were unsuitable for data extraction. Additionally, 2 records were identified through manual searching of other sources and were included in the eligibility assessment. Ultimately, 25 studies met the inclusion criteria and were included in the systematic review. We removed a study of high risk of bias; consequently, 24 papers were included in the meta-analysis (Figure 1).

**Figure 2:**
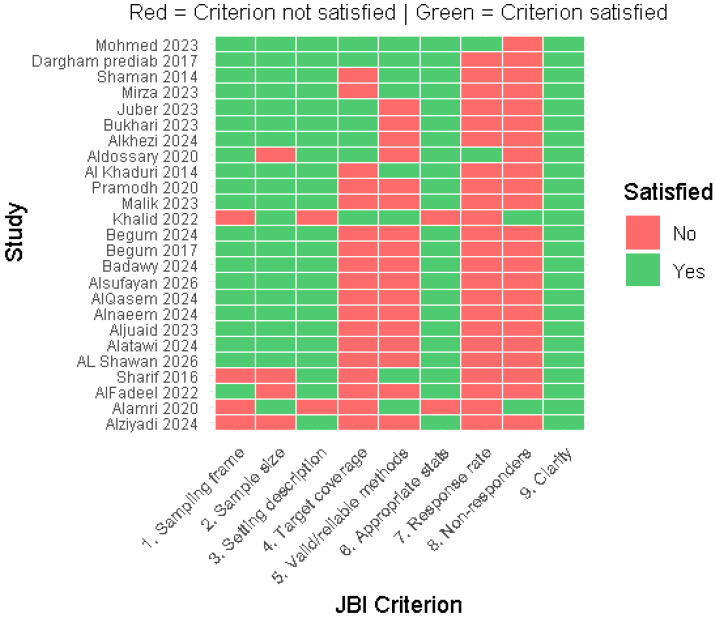
Joanna Briggs Institute checklist item performance per study (n = 25). Studies included were AlQasem 2024 [32], Aldossary 2020 [28], Mohmed 2023 [29], Malik 2023 [33], Alatawi 2024 [34], AlFadeel 2022 [35], Aljuaid 2023 [36], Bukhari 2023 [37], Begum 2024 [22], Begum 2017 [38], Sharif 2016 [39], Alkhezi 2024 [21], Al Khaduri 2014 [40], Juber 2023 [41], Al-ziyadi 2024 [42], Badawy 2024 [43], Alnaeem 2024 [44], Alsufayan 2026 [45], Al Shawan 2026 [20], Shaman 2014 [46], Dargham 2017 [47], Pramodh 2020 [10], Mirza 2023 [19], Alamri 2020 [30], and Khalid 2022 [31].

Item level: All studies (100%) demonstrated clarity of findings, indicating transparent reporting of objectives and results [Setting description and appropriate statistical analysis were also strong, each satisfied by 92.0% of studies. Appropriate sampling frame and adequate sample size were each met by 84.0% of studies. However, major deficiencies were observed in several critical domains: response rate reporting was provided by only 8.0% of studies (just 2 studies: Aldossary 2020 [28] and Mohmed 2023 [29]), non-responder characterization was also reported by only 8.0% (Alamri 2020 [30] and Khalid 2022 [31]), and valid/reliable measurement methods (e.g., using validated diagnostic criteria with biochemical or ultrasound confirmation) were satisfied by only 32.0% of studies. Target population coverage was also poor, satisfied with just 28.0% of studies, as shown in Figure 2.

**Figure 3:**
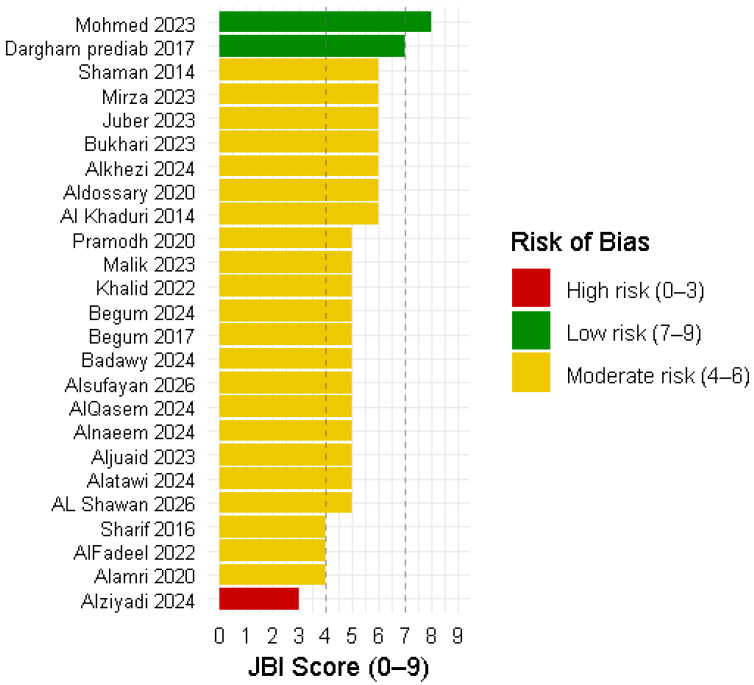
Joanna Briggs Institute quality assessment PMOS prevalence studies in Gulf countries (n = 25). Studies included were AlQasem 2024 [32], Aldossary 2020 [28], Mohmed 2023 [29], Malik 2023 [33], Alatawi 2024 [34], AlFadeel 2022 [35], Aljuaid 2023 [36], Bukhari 2023 [37], Begum 2024 [22], Begum 2017 [38], Sharif 2016 [39], Alkhezi 2024 [21], Al Khaduri 2014 [40], Juber 2023 [41], Al-ziyadi 2024 [42], Badawy 2024 [43], Alnaeem 2024 [44], Alsufayan 2026 [45], Al Shawan 2026 [20], Shaman 2014 [46], Dargham 2017 [47], Pramodh 2020 [10], Mirza 2023 [19], Alamri 2020 [30], and Khalid 2022 [31].

**Study level**: The vast majority (22 studies, 88.0%) fell into the moderate risk category (scores 4–6), while two studies (8.0%) achieved low risk status (scores 7–9) and one study (4.0%) was classified as high risk (score ≤ 3). Specifically, Mohmed 2023 [29] achieved the highest score of 8, followed by Dargham 2017 [47] with a score of 7, making them the only two studies with low risk of bias. The moderate risk group encompassed a wide distribution: 12 studies scored 5 (the most common score): AlQasem 2024 [32], Malik 2023 [33], Alatawi 2024 [34], Aljuaid 2023 [36], Begum 2024 [22], Begum 2017 [38], Pramodh 2020 [10], Badawy 2024 [43], Alnaeem 2024 [44], AL Shawan 2026 [20], Alsufayan 2026 [45], and Khalid 2022 [31]. Seven studies scored 6: Aldossary 2020 [28], Bukhari 2023 [37], Alkhezi 2024 [21], Al Khaduri 2014 [40], Juber 2023 [41], Mirza 2023 [19], and Shaman 2014 [46]. Three studies scored 4 (lower end of moderate risk): AlFadeel 2022 [35], Sharif 2016 [39], and Alamri 2020 [30]. The single high-risk study was Alziyadi 2024 [42], which scored 3. Figure 3.

By country, Saudi Arabia contributed the largest number of studies (n = 16, 65.4%), with a mean JBI score of 5.1 ± 1.1, ranging from 3 to 8. Saudi Arabia also had the only high-risk study (Alziyadi 2024 [42]) and one of the two low-risk studies (Mohmed 2023 [29]). Qatar (n = 2) had a mean score of 5.5 ± 2.1 but wide variability (range 4–7), including the other low-risk study (Dargham 2017 [47]) and one study at the lower moderate range (Sharif 2016 [39], score 4). The UAE (n = 4) and Oman (n = 2) had mean scores of 5.5 ± 0.6 and 5.5 ± 0.7 respectively, with all their studies in the moderate risk category (scores 5–6). Kuwait contributed a single study (Alkhezi 2024 [21]) scoring 6, placing it entirely in the moderate risk category. Figure 3.

**Qualitative synthesis of the study results**

Demographic criteria (Table 2)

The dataset includes 25 studies conducted across five GCC, with a clear predominance from Saudi Arabia (n = 16 studies), followed by the UAE (n = 4), Oman (n = 2), Qatar (n = 2), and Kuwait (n = 1). The study settings were university-based (particularly medical and health science students), general population surveys, hospital and tertiary care clinics, infertility clinics, and population-level registries. The age range across studies focuses predominantly on reproductive-aged women, typically 18–30 years for university-based samples, with some extending to 45–50 years. Sampling frames differ markedly (student-based convenience samples versus population-based and clinical registries), as do diagnostic criteria, participant demographics, and recruitment strategies. Sample sizes also vary widely, ranging from small university cohorts (100) to very large population datasets exceeding 64,000 participants. Reported PMOS prevalence ranged from as low as 1.6% [19] in a large hospital-based UAE dataset to as high as 56% [31] in a Saudi infertility clinic population. Among general or university populations using self-report or physician diagnosis, prevalence typically falls between 13% and 27%.

Diagnostic criteria & clinical features (Table 3)

Considerable heterogeneity was observed in diagnostic approaches across studies. The majority relied on self-reported questionnaires or self-reported physician diagnosis. A smaller subset employed more standardized diagnostic frameworks, including the Rotterdam criteria, the NIH criteria, or combined clinical, biochemical, and ultrasound-based assessments. Studies applying stricter diagnostic protocols such as Rotterdam-based evaluations (e.g., Al Khaduri 2014 [40], Mohmed 2023 [29]) or NIH criteria generally reported moderate prevalence estimates (approximately 7–19%). In contrast, studies relying on symptom-based self-reporting frequently reported wider variability and, in some cases, higher prevalence estimates. Notably, biochemical confirmation was not consistently applied across studies, and only a limited number incorporated comprehensive hormonal profiling alongside imaging. Ultrasound-based confirmation was also inconsistently used, further contributing to diagnostic variability. Clinical manifestations of PMOS varied widely across studies but were generally consistent with established phenotypes, including menstrual irregularity, hirsutism, acne, alopecia, and metabolic features such as weight gain and obesity. However, reporting was inconsistent, with several studies omitting key clinical indicators. Menstrual irregularity was among the most frequently reported symptoms, ranging from 13.2% [45] to 100% [46,47] in clinically confirmed cohorts. Hirsutism and acne prevalence also showed broad variability, with some studies reporting rates exceeding 70% in clinically diagnosed populations, while others reported much lower figures in self-reported samples. Alopecia and acanthosis nigricans were less consistently reported but, when included, were notably more frequent in clinically or symptom-based cohorts. BMI reporting was inconsistent: many studies did not report mean BMI, only noting the percentage overweight or obese. Among those that did, PMOS groups consistently showed higher BMI or obesity prevalence, with overweight/obesity rates ranging from 17% (Alziyadi 2024 [42]) to 72.6% (Mohmed 2023 [29]) in PMOS cases.

Metabolic Outcomes, Comorbidities & Awareness (Table 4)

Family history of PMOS was inconsistently reported across studies but, where available, ranged from 11.6% to 64.0%. Several studies also reported high prevalence of family history of related metabolic disorders, particularly diabetes, further supporting a shared genetic and environmental susceptibility to metabolic dysfunction in affected populations. Metabolic comorbidities were commonly reported among women with PMOS, although the frequency varied considerably depending on study design and population type. Diabetes or dysglycemia was reported in multiple studies, ranging from low prevalence estimates (e.g., 0.4–4.3% in some community-based samples) to substantially higher rates in hospital- or high-risk cohorts (e.g., up to 45.9% in Aljuaid 2023 [36]). Prediabetes and impaired glucose regulation were also frequently noted, including elevated proportions in studies such as Dargham 2017 [47] (19.4%) and Mohmed 2023 [29]. Hypertension or elevated blood pressure was less consistently reported but was present in several cohorts, with evidence of both prehypertension and clinically elevated blood pressure in PMOS populations. Dyslipidemia, particularly low high-density lipoprotein levels, was also observed in a subset of studies. Metabolic syndrome or clustered cardiometabolic risk was explicitly reported in at least one study, with Shaman 2017 [46] documenting a prevalence of 58%. Additional studies further supported this association through elevated odds ratios linking PMOS with metabolic abnormalities (e.g., Mohmed 2023 [29], odds ratio = 4.62).

Reproductive outcomes were also commonly affected. Infertility was variably reported, ranging from approximately 7.5% in general or mixed populations (AlQasem 2024 [32]) to as high as 70–90% in clinical or infertility-focused cohorts (e.g., Alatawi 2024 [34], Aljuaid 2023 [36]). In specialized fertility clinics, primary and secondary infertility patterns were frequently observed, further emphasizing the reproductive burden of PMOS in clinical settings. Anxiety, depression, and psychological distress were frequently observed, with prevalence estimates ranging from approximately 31% to over 60% in some cohorts (e.g., Bukhari 2023 [37], Alnaeem 2024 [44]). One study reported a particularly high burden of mental health symptoms (91% in Aljuaid 2023 [36]). Awareness of PMOS varied widely across studies. Reported awareness ranged from very low levels of adequate knowledge (e.g., 2.9% good knowledge in Bukhari 2023 [37] to extremely high awareness levels in selected university samples (up to 99% in Aldossary 2020 [28]). However, most studies reported moderate awareness levels between approximately 38% and 83%. One study specifically assessed dietary awareness and reported high levels (93.1%) [45].

**Table 2:** Study Characteristics (Demographics & Prevalence).

Study	Country & Nationality	Setting	Age	Marital Status	Education/Population	Employment	N/PMOS Cases/Prevalence
AlQasem 2024 [32]	Saudi (Abha)/Saudi	University	18–29	84.5% single, 14% married	Medical students	Students	200/53/26.5%
Aldossary 2020 [28]	Saudi (Riyadh)/Saudi	University	21–25	NR	Pharmacy students	Students	100/16/16.0%
Mohmed 2023 [29]	Saudi (Riyadh)/Saudi	Hospital	18–30	NR	NR	NR	1080/204/18.9%
Malik 2023 [33]	Saudi (Jazan)/Saudi	General	18–50	54.5% single, 45.5% married	79.6% undergrad	NR	363/54/15.0%
Alatawi 2024 [34]	Saudi (Tabuk)/97.1% Saudi	General	18–24	52.6% single, 40.4% married	64.8% bachelor’s	41.7% students	384/56/14.6%
AlFadeel 2022 [35]	Saudi (Riyadh)/Saudi	University	21–30	NR	Medical students	Students	216/42/19.0%
Aljuaid 2023 [36]	Saudi (national)/93.9% Saudi	General	18–45	55.6% married, 39.3% single	72% bachelor’s	21.1% healthcare workers	379/178/46.9%
Bukhari 2023 [37]	Saudi (Western)/Saudi	General	20–30	51.9% single, 42.3% married	46.4% bachelor’s	NR	418/133/31.8%
Begum 2024 [22]	Oman/Omani	University	18–21	NR	Medical students	Students	154/7/4.6%
Begum 2017 [38]	UAE/Emirati + others	University	18–24	NR	Medical students	Students	250/69/27.6%
Sharif 2016 [39]	Qatar/Qatari	University	18–30	NR	University students	Students	120/22/18.3%
Alkhezi 2024 [21]	Kuwait/86.5% Kuwaiti	University	18–24	95.6% single	Health sciences students	Students	588/96/16.3%
Al Khaduri 2014 [40]	Oman/Omani	Hospital	15–45	NR	NR	NR	3644/255/7.0%
Juber 2023 [41]	UAE/Emirati	Population	25–67	95.6% single	NR	NR	1040/269/25.9%
Alziyadi 2024 [42]	Saudi (Taif)/Saudi	Medical students	NR	NR	Medical students	Students	243/23/9.5%
Badawy 2024 [44]	Saudi (Jeddah)/Saudi	Medical students	20–22	NR	Medical students	Students	272/22/8.1%
Alnaeem 2024 [44]	Saudi (national)/97% Saudi	General	26–35	49% married, 46% single	NR	NR	967/367/37.9%
Alsufayan 2026 [45]	Saudi (Riyadh)/Saudi	Medical students	18–24	98.7% single	Medical students	Students	303/56/18.5%
AL Shawan 2026 [20]	Saudi (Eastern)/Saudi	General	21–29	52.8% married, 42.4% single	49.5% university	NR	408/81/19.9%
Shaman 2017 [46]	Saudi (Tabuk)/Saudi	Hospital (infertile)	15–45	NR	NR	NR	404/76/18.8%
Dargham prediab 2017 [47]	Qatar/Qatari	Population	18–40	NR	NR	NR	720/87/12.1%
Pramodh 2020 [10]	UAE/Emirati	University	18–25	92% single	University students	Students	493/64/13.0%
Mirza 2023 [19]	UAE (Dubai)/Emirati	Hospital	15–45	NR	NR	NR	64,722/1031/1.6%
Alamri 2020 [30]	Saudi (Arar)/Saudi	Infertility hospital	30–40	Married	NR	NR	565/123/21.8%
Khalid 2022 [31]	Saudi (Najran)/Saudi	Hospital (infertility)	35–44	NR	NR	NR	100/56/56.0%

NR = not reported; N = total sample size; PMOS cases = number of participants diagnosed or reporting polycystic ovary syndrome; prevalence calculated as reported in each study; population categories (e.g., students, general population, hospital-based) reflect original study sampling frames; nationality refers to study-reported or assumed majority population when specified. In this paper, there was a discrepancy in cases with PMOS.

**Table 3:** Diagnostic Criteria & Clinical Features of study participants diagnosed with PMOS in GCC.

Study	Diagnostic Criteria	Method	Menstrual Irregularity	Hirsutism	Acne	Alopecia	Acanthosis Nigricans	Obesity/Weight Gain
AlQasem 2024 [32]	Self-report	Questionnaire	23% oligo	23%	53.50%	75.50%	41%	43.5% weight gain
Aldossary 2020 [28]	Self-report	Questionnaire	23%	NR	NR	22%	NR	28% obese/overweight PMOS
Mohmed 2023 [29]	Rotterdam	Clinical + US + hormones	17.60%	6.90%	NR	17.50%	NR	72.6% obese/overweight
Malik 2023 [33]	Self-report	Questionnaire	NR	NR	NR	NR	NR	NR
Alatawi 2024 [34]	Self-report	Questionnaire	86.70%	70.10%	67.70%	NR	NR	NR
AlFadeel 2022 [35]	Self-report	Questionnaire	41% abnormal, 31% irregular	35%	17%	28%	NR	NR
Aljuaid 2023 [36]	Medical diagnosis	Clinical	92.90%	85.50%	68.60%	75.70%	NR	39.8% overweight
Bukhari 2023 [37]	Self-report	Questionnaire	34.90%	19.90%	32.80%	NR	NR	33.30%
Begum 2024 [22]	NIH	Questionnaire	23.30%	23.30%	70.10%	NR	NR	3.9% overweight
Begum 2017 [38]	NIH	Questionnaire	100%	56.50%	49.30%	NR	NR	15.9% obese
Sharif 2016 [39]	NIH	Clinical + biochemical	100%	100%	63.60%	NR	NR	40.9% obese
Alkhezi 2024 [21]	Physician diagnosis	Self-report	36.7% infrequent	32.30%	74%	26.40%	39.60%	43% weight gain
Al Khaduri 2014 [40]	Rotterdam	Clinical + US + hormones	100%	NR	NR	NR	NR	25% BMI > 30
Juber 2023 [41]	Self-report	Questionnaire	NR	NR	NR	NR	NR	NR
Alziyadi 2024 [42]	Self-report	Questionnaire	17.40%	NR	64.9% acne/oily	NR	14.90%	17% overweight
Badawy 2024 [43]	Self-report	Questionnaire	NR	5%	36%	6%	22%	30.5% obese/overweight
Alnaeem 2024 [44]	Self-report	Questionnaire	NR	NR	NR	NR	NR	NR
Alsufayan 2026 [45]	Physician diagnosis	Questionnaire	13.2% oligo	22.80%	49.80%	60.70%	19.80%	32% weight gain
AL Shawan 2026 [20]	Symptom-based	Questionnaire	29.90%	38.50%	45.60%	45.10%	44.10%	27.20%
Shaman 2017 [46]	Rotterdam/NIH	Clinical + biochemical	100%	NR	NR	NR	NR	68% obese
Dargham prediab 2017 [47]	NIH	Hormones + history	100%	NR	NR	NR	NR	Elevated BMI
Pramodh 2020 [10]	Physician diagnosis	Questionnaire	30.70%	24%	40.60%	NR	13.50%	NR
Mirza 2023 [19]	ICD-10	EMR	NR	NR	NR	NR	NR	61.3% obese/overweight
Alamri 2020 [30]	Clinical + US	Clinical	74%	NR	NR	NR	NR	39.3% obese
Khalid 2022 [31]	Ultrasound	Endovaginal + transabdominal	NR	NR	NR	NR	NR	NR

NR = Not reported; mFG = Modified Ferriman–Gallwey score; US = Ultrasound; NIH = National Institutes of Health criteria; Rotterdam = Rotterdam diagnostic criteria for PMOS (requires ≥2 of oligo/anovulation, hyperandrogenism, or polycystic ovarian morphology); Clinical + biochemical diagnosis = Hormonal and imaging confirmation used together; Self-report = Participant-reported diagnosis or questionnaire-based assessment; Menstrual irregularity = Includes oligo/amenorrhea, irregular cycles, or abnormal bleeding patterns as defined in each study; Acne, hirsutism, alopecia, and acanthosis nigricans = Reported prevalence (%) when available; EMR = Electronic medical records; ICD-10 = International Classification of Diseases, 10th revision.

**Quantitative synthesis**

**Figure 4:**
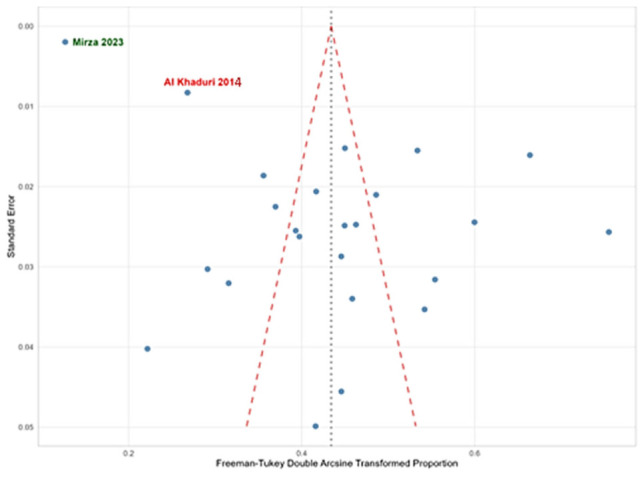
The funnel plot assessed publication bias among studies included in the meta-analysis of PMOS prevalence in Gulf countries. The x-axis represents the Freeman–Tukey double arcsine transformed proportion, while the y-axis represents the standard error (inverted, with smaller values at the top indicating larger sample sizes and greater precision). Dashed red lines represent the pseudo 95% confidence limits around the pooled effect estimate, forming a funnel-shaped region within which 95% of studies would be expected to fall in the absence of publication bias. Mirza (2023) [19] and Al Khaduri (2014) [40] were positioned on the left side of the funnel and outside the main cluster of studies, suggesting potential outlying influence and contributing to observed heterogeneity.

**Publication bias:** The funnel plot shows that all studies are located on the left side of the plot, with transformed proportions ranging from approximately 0.13 to 0.76. The plot demonstrates reasonable symmetry around the central pooled estimate. Larger studies (with standard errors near zero) are clustered near the top of the plot, while smaller studies (with higher standard errors) are scattered more widely at the bottom. Figure 4 Using the metafor package, Egger’s test was not significant (z = −0.5942, *p* = 0.5524) while Begg’s test was significant (Kendall’s tau = −0.3696, *p* = 0.0111), indicating asymmetry, as shown in Figure 4.

**Figure 5:**
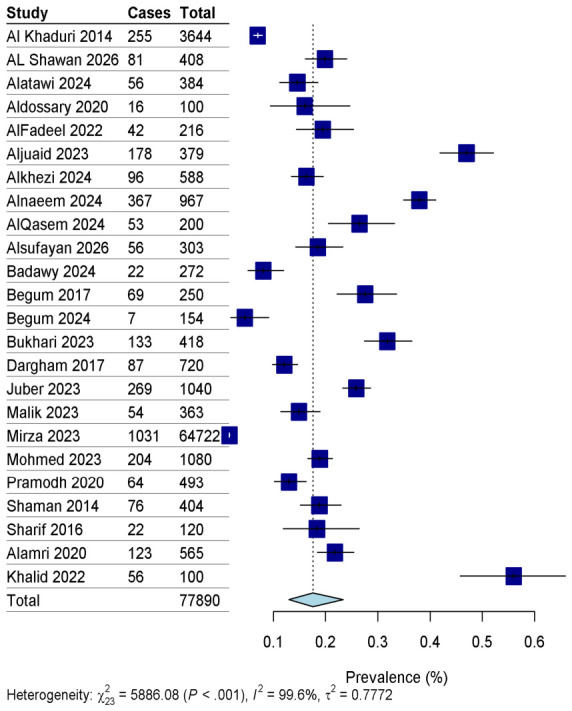
Overall pooled prevalence of PMOS in Gulf Cooperation Council countries: A random effects meta-analysis (n = 24 studies). The plot displays individual study prevalence estimates with 95% confidence intervals (blue squares), the overall pooled estimate under the random effects model (diamond), and heterogeneity statistics. Studies included were AlQasem 2024 [32], Aldossary 2020 [28], Mohmed 2023 [29], Malik 2023 [33], Alatawi 2024 [34], AlFadeel 2022 [35], Aljuaid 2023 [36], Bukhari 2023 [37], Begum 2024 [22], Begum 2017 [38], Sharif 2016 [39], Alkhezi 2024 [21], Al Khaduri 2014 [40], Juber 2023 [41], Alziyadi 2024 [42], Badawy 2024 [43], Alnaeem 2024 [44], Alsufayan 2026 [45], Al Shawan 2026 [20], Shaman 2014 [46], Dargham 2017 [47], Pramodh 2020 [10], Mirza 2023 [19], Alamri 2020 [30], and Khalid 2022 [31].

The forest plot presents the meta-analysis of 24 studies examining the prevalence of PMOS among women in GCC, comprising a total of 77,890 participants and 3417 reported PMOS cases. Individual study prevalence estimates varied substantially across studies, ranging from 1.6% [19] to 47.0% [36]. Mirza 2023, which contributed the largest sample size (N = 64,722), reported the lowest prevalence estimate and exhibited the narrowest confidence interval. In contrast, smaller studies such as Aldossary 2020 [28] (N = 100) and Begum 2024 [22] (N = 154) produced wider confidence intervals, indicating greater uncertainty around their prevalence estimates. The pooled effect size was 17.59% (95% CI: 12.98–23.40%). The analysis revealed extremely high between-study heterogeneity. The I^2^ statistics were 99.6%. This considerable heterogeneity is visually evident in the forest plot, where prevalence estimates are widely dispersed across the range of reported values. Given this level of heterogeneity, a random-effects model was appropriately employed, as it accounts for both within-study and between-study heterogeneity and provides a more conservative pooled estimate than a fixed-effect approach. The relatively wide confidence interval surrounding the pooled prevalence estimate further reflects the uncertainty introduced by the substantial heterogeneity among studies, as shown in Figure 5.

**Table 4:** Metabolic Outcomes, Comorbidities & Awareness.

Study	Infertility	Diabetes	Hypertension	Dyslipidemia	Family History PMOS	Other Findings	Awareness
AlQasem 2024 [32]	7.50%	4% DM, 10% prediabetic	NR	NR	47%	Multiple symptoms	80%
Aldossary 2020 [28]	NR	NR	NR	NR	NR	NR	99%
Mohmed 2023 [29]	NR	NR	Pre-HTN/HTN	NR	NR	OR 4.62	19.00%
Malik 2023 [33]	NR	NR	NR	NR	NR	Weight perception	48.50%
Alatawi 2024 [34]	70.10%	42.20%	NR	42.20%	NR	Symptoms identified	83.30%
AlFadeel 2022 [35]	NR	NR	NR	NR	64.00%	Anxiety/depression	NR
Aljuaid 2023 [36]	90.20%	45.90%	NR	NR	29.80%	Mental health 91%	NR
Bukhari 2023 [37]	16.30%	30.10%	NR	30.60%	NR	Psychological 62.7%	2.9% good
Begum 2024 [22]	NR	NR	NR	NR	21.40%	NR	NR
Begum 2017 [38]	NR	NR	NR	NR	60.80%	Acne/hirsutism	NR
Sharif 2016 [39]	NR	95.5% family DM	NR	NR	40.90%	Acne/hirsutism	NR
Alkhezi 2024 [21]	NR	10.80%	11.50%	NR	26.30%	Hyperprolactinemia	NR
Al Khaduri 2014 [40]	NR	NR	NR	NR	NR	NR	NR
Juber 2023 [41]	NR	10.80%	26%	11.50%	NR	Asthma 17.5%	NR
Alziyadi 2024 [42]	NR	3.30%	NR	NR	11.60%	Anxiety 54.4%	46.90%
Badawy 2024 [43]	NR	0.40%	0.70%	2.60%	NR	Sleep & cognition issues	NR
Alnaeem 2024 [44]	NR	NR	NR	NR	31%	Depression/anxiety/stress	NR
Alsufayan 2026 [45]	NR	1% DM, 3% prediabetic	NR	NR	42.20%	Family PMOS	93.10%
AL Shawan 2026 [20]	NR	3.70%	NR	NR	17.90%	Psychological burden	83.10%
Shaman 2017 [46]	68%	15.80%	Yes	Low HDL	NR	Metabolic syndrome 58%	NR
Dargham prediab 2017 [47]	NR	9.70%	Elevated BP	Low HDL	NR	Prediabetes 19.4%	NR
Pramodh 2020 [10]	NR	4.30%	NR	NR	NR	Family DM 75.5%	38.30%
Mirza 2023 [19]	NR	NR	Elevated SBP/DBP	NR	NR	Age association	NR
Alamri 2020 [30]	Infertility cases	NR	NR	NR	NR	Ovulation defects	NR
Khalid 2022 [31]	Primary 65%, secondary 35%	NR	NR	NR	NR	Fibroids, polyps, etc.	NR

NR = not reported; DM = diabetes mellitus; HTN = hypertension; SBP/DBP = systolic/diastolic blood pressure; dyslipidemia includes any reported abnormal lipid profile (e.g., low HDL, elevated LDL, or general dyslipidemia); infertility refers to self-reported or clinically defined subfertility/infertility depending on study design; family history PMOS/DM refers to first-degree or any reported family history as defined by each study; awareness refers to participant knowledge or awareness of PMOS or its complications as measured in questionnaires; other findings include additional metabolic, psychological, or reproductive comorbidities reported by authors.

**Figure 6:**
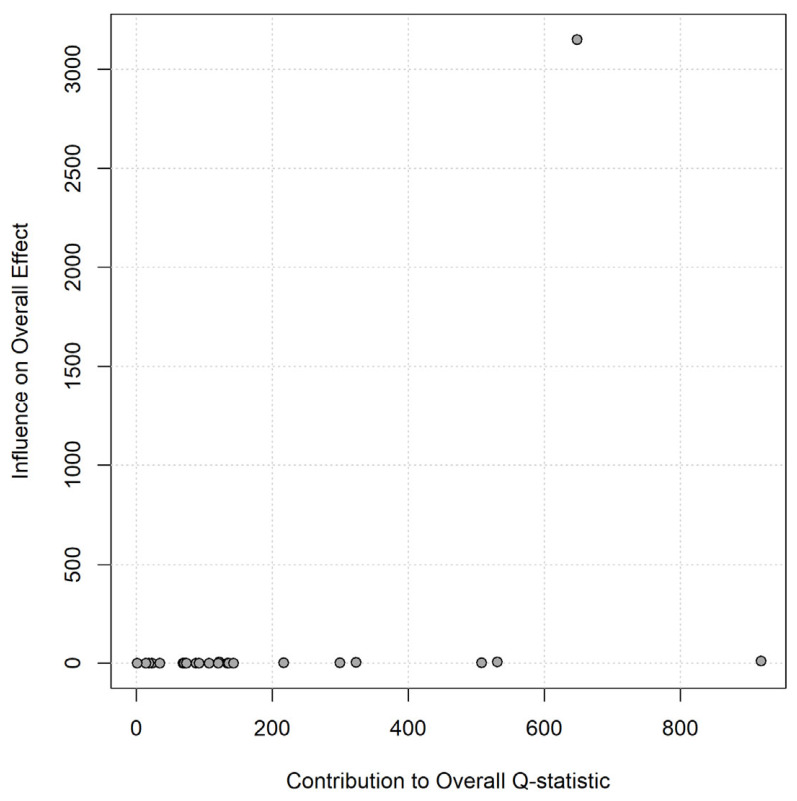
Identification of Outlier Studies Contributing to Heterogeneity in the Meta-Analysis of PMOS Prevalence in Gulf Countries.

Based on the Baujat plot, two studies were identified as statistical outliers contributing most to the observed heterogeneity. Mirza 2023 [19] is in the upper-right quadrant, demonstrating the highest contribution to the overall Q-statistics due to its extremely large sample size and very low prevalence which deviates substantially from most other studies with high effect on the pooled prevalence. Alnaeem 2024 [44] is in the lower-right quadrant, demonstrating both high contribution to heterogeneity and low influence on the pooled effect, with a prevalence of 38.0% that is notably above average. The presence of these two outliers explains a substantial portion of the considerable heterogeneity observed in the meta-analysis, as shown in Figure 6.

The leave-one-out sensitivity analysis demonstrated that the pooled prevalence remains highly stable across all 24 iterations, with estimates ranging narrowly from 16.53% to 19.41%, compared with the overall pooled prevalence of 17.59%. When Mirza 2023 [19] was excluded, the pooled prevalence increased to 19.41% (95% CI: 15.26–24.36%), which is the highest observed estimate across all iterations. This suggests that Mirza 2023 [19], despite its very large sample size, exerts a slight downward influence on the pooled estimate due to its relatively low prevalence compared with other included studies. Conversely, when lower-prevalence studies such as Khalid 2022 [31] (16.53%) and Aljuaid 2023 [36] (16.70%) were removed, the pooled prevalence decreases to approximately 16.5–16.7%, indicating their modest upward influence on the pooled estimate. Similarly, exclusion of most other studies results in pooled estimates clustered tightly between 17.0% and 18.2%, further confirming the robustness of the overall prevalence estimate. Across all iterations, heterogeneity remains extremely high and stable (I^2^ = 99.6%), indicating persistent and substantial between-study variability regardless of which study is removed. The between-study variance (τ^2^) also shows minimal fluctuation across iterations, generally remaining around 0.67 to 0.81, with only small reductions when individual studies are excluded. The Q-test remains highly significant in all scenarios (*p* < 0.001), confirming that heterogeneity is not driven by any single study but is instead intrinsic to the dataset, as shown in Table 5.

**Figure 7:**
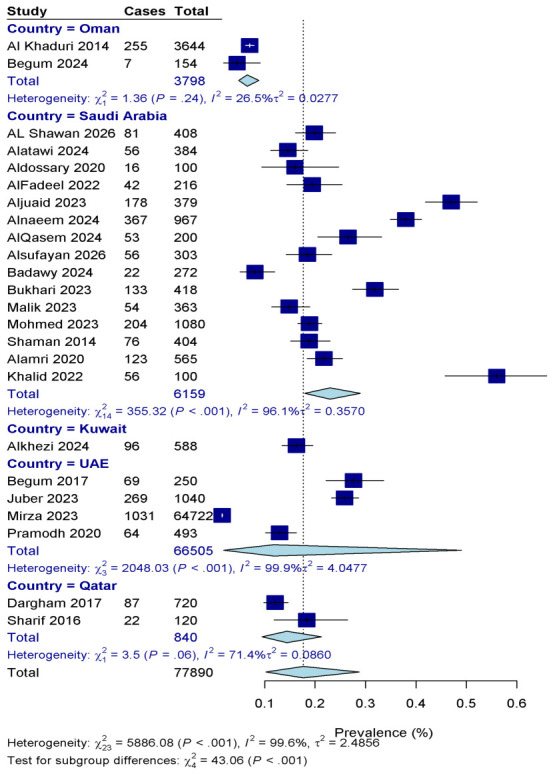
The pooled prevalence estimates stratified by country. Studies included were AlQasem 2024 [32], Aldossary 2020 [28], Mohmed 2023 [29], Malik 2023 [33], Alatawi 2024 [34], AlFadeel 2022 [35], Aljuaid 2023 [36], Bukhari 2023 [37], Begum 2024 [22], Begum 2017 [38], Sharif 2016 [39], Alkhezi 2024 [21], Al Khaduri 2014 [40], Juber 2023 [41], Alziyadi 2024 [42], Badawy 2024 [43], Alnaeem 2024 [44], Alsufayan 2026 [45], Al Shawan 2026 [20], Shaman 2014 [46], Dargham 2017 [47], Pramodh 2020 [10], Mirza 2023 [19], Alamri 2020 [30], and Khalid 2022 [31].

Figure 7 presents the pooled prevalence estimates stratified by country. Oman showed the lowest pooled prevalence at 6.56% (95% CI: 4.84–8.83%), with low-to-moderate heterogeneity between studies (I^2^ = 26.5%). Saudi Arabia demonstrated the highest pooled prevalence at 23.0% (95% CI: 17.94–28.98%), accompanied by substantial heterogeneity (I^2^ = 96.1%). Kuwait was represented by a single study, yielding a prevalence estimate of 16.33% (95% CI: 13.55–19.54%); therefore, heterogeneity could not be assessed. In the UAE, the pooled prevalence was 11.79% (95% CI: 1.82–49.05%), with extremely high heterogeneity (I^2^ = 99.9%). Similarly, Qatar showed a pooled prevalence of 14.4% (95% CI: 9.48–21.26%) and substantial heterogeneity (I^2^ = 71.4%).

**Table 5:** Leave-One-Out Sensitivity Analysis with Heterogeneity Assessment for PMOS Prevalence Meta-Analysis.

Study Excluded	Pooled Prevalence (%)	95% CI	I^2^ (%)	τ^2^	Q-Test *p*-Value
Al Khaduri 2014 [40]	18.28	13.47–24.32	99.6	0.7602	0.001
AL Shawan 2026 [20]	17.49	12.73–23.56	99.6	0.8116	0.001
Alatawi 2024 [34]	17.73	12.92–23.84	99.6	0.8097	0.001
Aldossary 2020 [28]	17.67	12.88–23.75	99.6	0.8092	0.001
AlFadeel 2022 [35]	17.51	12.75–23.58	99.6	0.8113	0.001
Aljuaid 2023 [36]	16.7	12.35–22.19	99.6	0.7181	0.001
Alkhezi 2024 [21]	17.65	12.85–23.75	99.6	0.8121	0.001
Alnaeem 2024 [44]	16.92	12.41–22.64	99.6	0.7605	0.001
AlQasem 2024 [32]	17.26	12.58–23.22	99.6	0.8001	0.001
Alsufayan 2026 [45]	17.55	12.78–23.63	99.6	0.8121	0.001
Badawy 2024 [43]	18.15	13.33–24.22	99.6	0.7758	0.001
Begum 2017 [38]	17.22	12.56–23.16	99.6	0.7973	0.001
Begum 2024 [22]	18.46	13.72–24.37	99.6	0.7259	0.001
Bukhari 2023 [37]	17.09	12.49–22.95	99.6	0.7845	0.001
Dargham prediab 2017 [47]	17.87	13.04–23.99	99.6	0.8031	0.001
Juber 2023 [41]	17.27	12.58–23.24	99.6	0.8017	0.001
Malik 2023 [33]	17.72	12.91–23.83	99.6	0.8101	0.001
Mirza 2023 [19]	19.41	15.26–24.36	97.6	0.4802	0.001
Mohmed 2023 [29]	17.53	12.76–23.61	99.6	0.8126	0.001
Pramodh 2020 [10]	17.82	13.00–23.94	99.6	0.806	0.001
Shaman 2017 [46]	17.54	12.76–23.61	99.6	0.8122	0.001
Sharif 2016 [39]	17.57	12.79–23.64	99.6	0.8107	0.001
Alamri 2020 [30]	17.42	12.68–23.46	99.6	0.8096	0.001
Khalid 2022 [31]	16.53	12.33–21.79	99.6	0.6745	0.001

Note: I^2^ values are presented as proportions (e.g., 1.00 = 100%, 0.99 = 99%, 0.98 = 98%). The overall pooled prevalence (all studies included) was approximately 17.59% with considerable heterogeneity (I^2^ ≈ 100%). The Q-statistic *p*-value remained <0.001 across all iterations. Tau^2^ represents the between-study variance, with lower values indicating greater consistency among studies.

**Figure 8:**
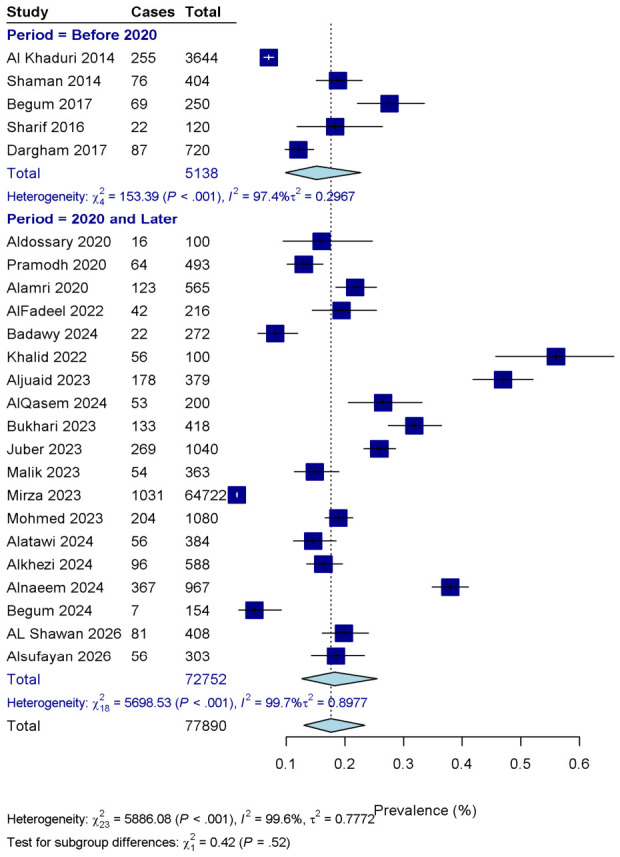
The pooled prevalence estimates stratified by period before vs. after 2020. Studies included were AlQasem 2024 [32], Aldossary 2020 [28], Mohmed 2023 [29], Malik 2023 [33], Alatawi 2024 [34], AlFadeel 2022 [35], Aljuaid 2023 [36], Bukhari 2023 [37], Begum 2024 [22], Begum 2017 [38], Sharif 2016 [39], Alkhezi 2024 [21], Al Khaduri 2014 [40], Juber 2023 [41], Alziyadi 2024 [42], Badawy 2024 [43], Alnaeem 2024 [44], Alsufayan 2026 [45], Al Shawan 2026 [20], Shaman 2014 [46], Dargham 2017 [47], Pramodh 2020 [10], Mirza 2023 [19], Alamri 2020 [30], and Khalid 2022 [31].

Figure 8 presents the pooled PMOS prevalence estimates between studies published before and after 2020. The pooled prevalence was 15.2% (95% CI: 9.9–22.7%) for the pre-2020 period (5 studies, I^2^ = 97.4%) and 18.2% (95% CI: 12.6–25.5%) for the post-2020 period (19 studies, I^2^ = 99.7%). The test for subgroup differences was not significant (Q-between = 0.42, *p* = 0.52), indicating no statistically significant change in PMOS prevalence over time. Despite a higher point estimate in more recent studies, the wide and overlapping confidence intervals, combined with very high heterogeneity within both periods (I^2^ > 97%), suggest that publication year does not explain the substantial variability in prevalence estimates.

**Figure 9:**
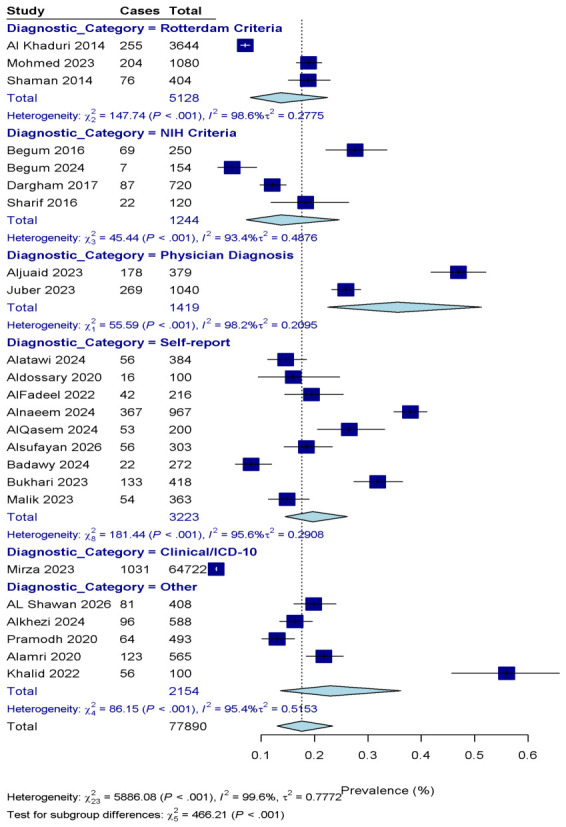
The pooled prevalence estimates stratified by diagnostic category. Studies included were AlQasem 2024 [32], Aldossary 2020 [28], Mohmed 2023 [29], Malik 2023 [33], Alatawi 2024 [34], AlFadeel 2022 [35], Aljuaid 2023 [36], Bukhari 2023 [37], Begum 2024 [22], Begum 2017 [38], Sharif 2016 [39], Alkhezi 2024 [21], Al Khaduri 2014 [40], Juber 2023 [41], Alziyadi 2024 [42], Badawy 2024 [43], Alnaeem 2024 [44], Alsufayan 2026 [45], Al Shawan 2026 [20], Shaman 2014 [46], Dargham 2017 [47], Pramodh 2020 [10], Mirza 2023 [19], Alamri 2020 [30], and Khalid 2022 [31].

Figure 9 compares pooled PMOS prevalence estimates by assessment/diagnostic tool. The pooled prevalence varied substantially across methods, ranging from 1.59% using ICD-10 codes to 37.08% using ultrasound/clinical assessment. Rotterdam Criteria yielded a pooled prevalence of 13.7% (95% CI: 7.9–22.5%, I^2^ = 98.6%, 3 studies), while NIH Criteria produced 13.7% (95% CI: 7.2–24.7%, I^2^ = 93.4%, 4 studies). Self-report (9 studies) demonstrated a higher pooled prevalence of 19.7% (95% CI: 14.5–26.1%, I^2^ = 95.6%). Physician diagnosis (2 studies) yielded 35.6% (95% CI: 22.4–51.3%, I^2^ = 98.2%). The test for subgroup differences was highly significant (Q-between = 466.21, *p* < 0.001), confirming that diagnostic criteria significantly affect prevalence estimates.

**Figure 10:**
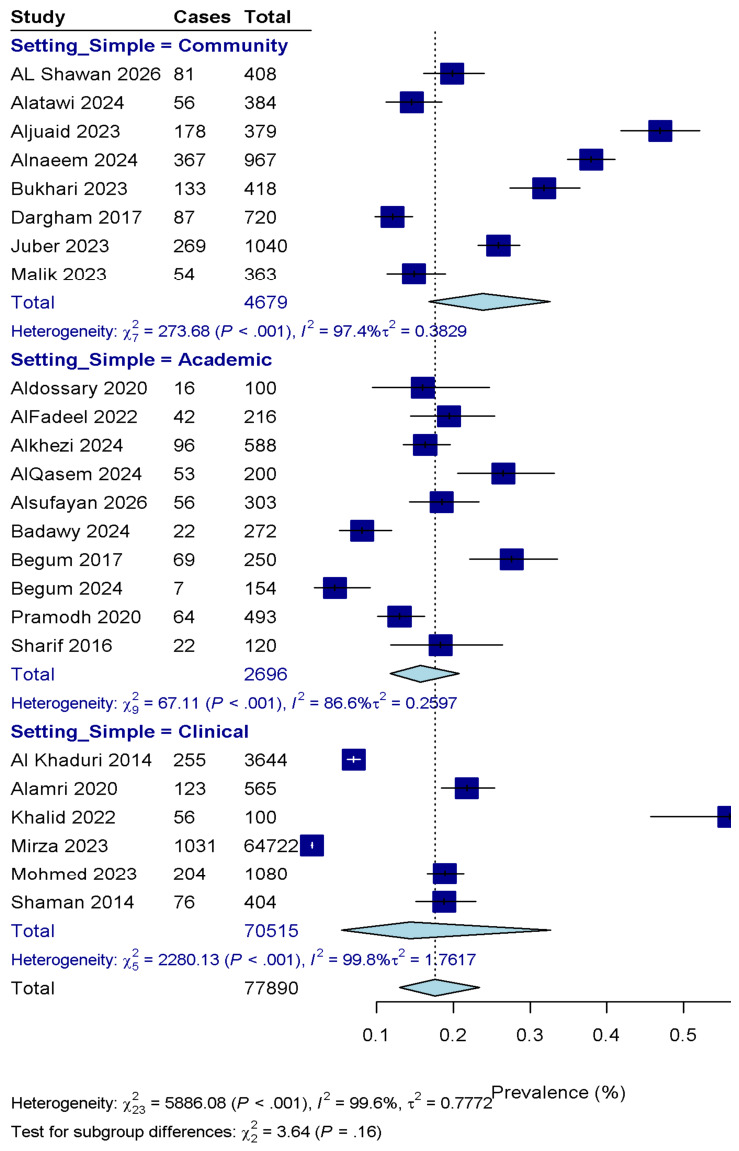
The pooled prevalence estimates stratified by setting. Studies included were AlQasem 2024 [32], Aldossary 2020 [28], Mohmed 2023 [29], Malik 2023 [33], Alatawi 2024 [34], AlFadeel 2022 [35], Aljuaid 2023 [36], Bukhari 2023 [37], Begum 2024 [22], Begum 2017 [38], Sharif 2016 [39], Alkhezi 2024 [21], Al Khaduri 2014 [40], Juber 2023 [41], Alziyadi 2024 [42], Badawy 2024 [43], Alnaeem 2024 [44], Alsufayan 2026 [45], Al Shawan 2026 [20], Shaman 2014 [46], Dargham 2017 [47], Pramodh 2020 [10], Mirza 2023 [19], Alamri 2020 [30], and Khalid 2022 [31].

Figure 10 compares pooled PMOS prevalence estimates across study settings. The pooled prevalence varied across settings, ranging from 14.0% in clinical settings to 24.0% in community-based studies. Community-based studies (8 studies) yielded the highest pooled prevalence at 23.8% (95% CI: 16.80–32.60%), I^2^ = 97.4%, followed by academic settings (10 studies) with a pooled prevalence of 15.7% (95% CI: 11.70–20.80%), I^2^ = 86.6%. Clinical settings (6 studies) demonstrated the lowest pooled prevalence at 14.3% (95% CI: 5.40–32.70%), I^2^ 99.8% Test for subgroup differences was not significant (Q-between = 3.64, *p* = 0.162).

A meta-regression analysis was conducted to explore sources of substantial heterogeneity. The assessment tool used for PMOS diagnosis was the most important moderator, explaining 42.7% of the between-study variance (QM = 21.01, df = 5, *p* = 0.0008). Compared to the Rotterdam/NIH criteria (reference: 13.9%, 95% CI: 8.7–21.4%), ultrasound/clinical assessment produced significantly higher prevalence (estimate: 1.28, 95% CI: 0.18–2.38, *p* = 0.022), with a predicted prevalence of 36.8% (95% CI: 10.3–74.6%). Conversely, ICD-10 codes produced significantly lower prevalence (estimate: −2.30, 95% CI: −3.73 to −0.87, *p* = 0.002), with a predicted prevalence of only 1.6% (95% CI: 0.2–10.2%). Self-report (20.3%) and physician diagnosis (22.9%) did not differ significantly from the reference (*p* > 0.05). The model containing only assessment tool showed improved fit compared to the null model (AIC: 52.44 vs. 64.98; BIC: 58.67 vs. 67.25). Study setting also significantly contributed to heterogeneity (QM = 11.35, df = 3, *p* = 0.010), explaining 27.8% of the variance. Descriptive analysis showed that clinical (hospital-based) settings had the lowest mean prevalence (11.6%, range: 1.6–18.9%), while community (21.1%, range: 12.1–37.9%) and academic settings (21.4%, range: 4.6–56.0%) demonstrated higher and more variable estimates. Country of origin was not a statistically significant moderator (QM = 7.85, df = 4, *p* = 0.097), explaining only 14.2% of the between-study variance. Although descriptive analysis revealed variation across countries, with Saudi Arabia showing the highest mean prevalence (24.6%, range: 7.0–56.0%) and Oman the lowest (5.8%, range: 4.6–7.0%), these differences did not reach statistical significance in the meta-regression model. The country model showed moderate improvement in fit (AIC: 59.35) compared to the null model (64.98). The combined model including both assessment tool and country explained the largest proportion of heterogeneity (R^2^ = 49.2%, QM = 30.16, df = 9, *p* = 0.0004) and demonstrated the best fit model (AIC: 49.99, BIC: 57.02). Table 6.

In the separate assessment tool model, ultrasound/clinical assessment showed significantly higher prevalence (β = 1.28, *p* = 0.022) and ICD-10 codes significantly lower prevalence (β = −2.30, *p* = 0.002) compared to the Rotterdam/NIH reference, while self-report and physician diagnosis were not significant. In the separate setting model, clinical settings showed significantly lower prevalence than community settings (β = −1.27, *p* = 0.006); academic settings and the “Other” category were not significant, though the latter should be interpreted with caution as it represents only one study. In the separate country model, no individual country differed significantly from Kuwait, consistent with the non-significant overall country model (*p* = 0.097). In the combined model (assessment tool + country), after adjusting for both moderators simultaneously, only ICD-10 code remained statistically significant (β = −2.75, *p* = 0.001). Ultrasound/clinical assessment was no longer significant (*p* = 0.247). None of the country’s coefficients reached significance in the combined model, as shown in Table 7.

## 4. Discussion

This systematic review and meta-analysis attempted to characterize the burden of PMOS across GCC countries, drawing on 24 studies and data from nearly 78,000 women. The pooled prevalence of PMOS of 17.59% suggests that PMOS affects a substantially larger proportion of GCC women like global estimates of 6–20% would predict [1,48]. Comparative evidence from previous meta-analyses highlights substantial variability in global prevalence estimates depending on diagnostic criteria. Neven et al. [49] reported a global PMOS prevalence of 12.1% (95% CI: 9.8–14.8; I^2^: 98.8%) using Rotterdam criteria, 7.9% (95% CI: 6.2–9.9; I^2^: 96.2%) using NIH criteria, 12.7% (95% CI: 8.2–17.9; I^2^: 98.0%) using AE-PCOS criteria, and 7.8% (95% CI: 5.8–10.0; I^2^: 99.4%) based on self-reported diagnosis. Similarly, Salari et al. [50] estimated a global prevalence of 9.2% (95% CI: 6.8–12.5%), with subgroup analyses showing 5.5% (95% CI: 3.9–7.7%) using NIH criteria, 11.5% (95% CI: 6.6–19.4%) using Rotterdam criteria, 7.1% (95% CI: 2.3–20.2%) using AE-PCOS criteria, and 11% (95% CI: 5.2–21.8%) based on self-report. Regionally, Chiaffarino et al. [51] reported higher prevalence estimates in Europe and the United States, ranging from 6.2% (95% CI: 5.3–7.0) using NIH 1990 criteria to 19.5% (95% CI: 17.3–21.6) using European Society of Human Reproduction and Embryology (ESHRE)/American Society for Reproductive Medicine (ASRM) 2003 consensus (ESHRE/ASRM 2003) criteria, and 15.0% (95% CI: 12.9–17.1) using AES-PCOS criteria. In China, Wu et al. [52] estimated a PCOS prevalence of 10.01% (95% CI: 8.31–11.89%). These findings highlight substantial heterogeneity in PMOS prevalence globally, largely driven by differences in diagnostic definitions and study populations.

In this study, one of the most striking findings was the substantial heterogeneity across studies. Prevalence estimates ranged from as low as 1.6%, based on ICD-10 administrative data from a large hospital in the UAE, to 46.9% in a national Saudi Arabian study, with the highest estimate reaching 56.0% in a study conducted in Najran, Saudi Arabia [31,36,50]. This wide range in prevalence estimates is not unexpected, given the substantial differences in study populations, clinical settings, and diagnostic approaches across the included studies. However, it highlights an important methodological issue: the estimated prevalence of PMOS is highly dependent on how the condition is defined and measured. This observation was supported by the meta-regression analysis, which demonstrated that the assessment tool accounted for more than 42% of the between-study heterogeneity, substantially exceeding the explanatory contribution of either country or study setting. These findings suggest that diagnostic criteria and case ascertainment methods are major determinants of the reported prevalence of PMOS in the GCC region.

Studies using the Rotterdam or NIH diagnostic criteria, which incorporate combinations of clinical, biochemical, and ultrasonographic features, generally yielded prevalence estimates within a relatively moderate range. In contrast, studies based on self-reported diagnoses or physician diagnoses without standardized diagnostic protocols tended to report higher and more variable prevalence estimates. Conversely, the exceptionally low prevalence observed in the administrative dataset reported by Mirza et al. [19] likely reflects under-ascertainment resulting from incomplete diagnostic coding rather than a low disease burden, a well-recognized limitation of ICD-based surveillance systems [53]. On the other hand, many studies reported lower prevalence using self-reported data [49,50]. Self-reported PMOS data should be interpreted cautiously because they reflect perceived or previously communicated diagnoses rather than standardized clinical confirmation. As a result, they are highly influenced by respondents’ health status awareness, access to healthcare services, and health literacy. These findings highlight the substantial influence of diagnostic methodology on prevalence estimates. They further underscore the need for greater methodological standardization and harmonization of diagnostic criteria in future PMOS epidemiological research.

Country-Level Patterns: Saudi Arabia, which contributed the largest share of studies, also showed the highest pooled prevalence at 23.0%. This finding is plausible given the well-established links between PMOS and obesity, and the fact that overweight and obesity prevalence among Saudi women has been rising steadily in recent decades [54]. Oman, by contrast, showed the lowest pooled prevalence (6.56%) with relatively low heterogeneity between its two included studies, suggesting that Omani women may carry a lower burden or that the studies there were more methodologically homogeneous. The UAE showed extremely wide confidence intervals (1.82–49.05%), driven by the coexistence of the large administrative Mirza 2023 [19] dataset and smaller university-based studies, and should be interpreted with caution. Qatar and Kuwait were each represented by very few studies, making country-level conclusions premature. It is noteworthy that no studies from Bahrain met the inclusion criteria, leaving one GCC country entirely unrepresented in the current evidence base. This represents an important gap in the regional literature, particularly given Bahrain’s shared demographic, nutritional, and lifestyle transitions with neighboring GCC countries. Like other Gulf states, Bahrain has experienced rapid urbanization and a high burden of obesity and other noncommunicable disease risk factors, all of which are closely linked to PMOS development and progression [55]. The absence of population-based prevalence data from Bahrain therefore limits the completeness of regional estimates and highlights the need for future epidemiological studies in this setting.

Clinical Features: The clinical profile of PMOS in GCC women is broadly consistent with that reported in other populations, although several notable patterns emerge. Menstrual irregularity was nearly universal in studies that specifically recruited women with a confirmed PMOS diagnosis or those attending infertility clinics, reaching 100% in several reports. In contrast, substantially lower prevalence estimates (13–41%) were observed in general population and university-based samples. This marked discrepancy is most likely attributable to selection bias, as women presenting to clinical or infertility services are inherently more likely to experience and report reproductive symptoms such as menstrual dysfunction compared with individuals identified through population-based screening [56].

Hirsutism ranged from 5% to 100% across studies a spread so wide as to be almost uninterpretable without knowing the measurement tool used. Studies applying formal scoring systems such as the Ferriman–Gallwey scale reported lower rates than those relying on patient self-report, where cultural perceptions of what constitutes “excess” hair vary considerably. This is a particularly important consideration in the GCC context, where ethnic background and local grooming norms may affect both patient reporting and clinician assessment [57].

Acne and oily skin were frequently reported, with rates ranging from 17% to 74%. Interestingly, acne appeared more prominent than hirsutism in several young university-based cohorts, possibly reflecting age-related phenotypic expression or differences in awareness. Obesity, broadly defined, was present in anywhere from 17% to 81% of PMOS cases depending on the study, with most hospital-based studies reporting rates above 30–40%. This is consistent with global evidence linking adiposity with PMOS severity and with the regional context of rising obesity rates in GCC countries [13,14,15].

Family history of PMOS, where reported, ranged from 11.6% to 64%, suggesting a heritable component that is consistent with what genetic studies have shown globally [58,59]. However, these estimates are affected by recall bias, as participants may be unaware of or unable to accurately report a family history of PMOS, especially in contexts where awareness of the condition is limited and formal diagnosis is under-recognized.

**Awareness Gap**: Perhaps the most practically concerning finding in our review is the wide variability in PMOS awareness. Across studies that measured this, awareness ranged from under 3% [37] to nearly 99% [28]. The highest figure came from a highly selected Riyadh university sample and almost certainly overestimates awareness in the general population. More representative studies suggest that while many young women have heard of PMOS, deep understanding of its symptoms, causes, and long-term implications remains limited [20]. Only one study by Alsufayan 2026 [45] specifically examined dietary awareness, finding relatively high rates, but this too came from a student population with elevated health literacy. Closing this awareness gap is not solely an educational challenge; it also has direct clinical implications. Delayed recognition of PMOS may postpone diagnosis and management, thereby limiting opportunities for early lifestyle interventions that can meaningfully reduce long-term metabolic risk [60].

## 5. Strengths and Limitations

The strength of this review lies in its breadth, encompassing 24 studies across five GCC countries and nearly 78,000 participants, alongside a rigorous quality appraisal using validated JBI criteria. The sensitivity analyses further supported the robustness of the pooled estimate, as exclusion of any single study resulted in a change of no more than 2 percentage points. In addition, leave-one-out analyses demonstrated no disproportionate influence from any individual study, including the large administrative dataset reported by Mirza et al. (2023) [19]. Finally, the use of meta-regression provided additional insight into sources of heterogeneity, identifying diagnostic criteria and study setting as significant moderators of PMOS prevalence. However, heterogeneity was extreme across all analyses, meaning that the pooled prevalence figure should be understood as an approximation reflecting highly diverse study populations rather than a precise epidemiological parameter. Most included studies (88%) were rated as moderate quality, with key weaknesses in response rate reporting, non-responder characterization, and use of validated diagnostic instruments. Most studies relied on self-report or convenience sampling from university populations, which may overrepresent educated young women and underestimate PMOS burden among older, less educated, or rural women. Bahrain was entirely absent from the evidence base, and the number of studies from Oman, Qatar, and Kuwait was insufficient to draw firm country-level conclusions. Furthermore, publication bias could not be fully excluded. While Egger’s test was non-significant, Begg’s test suggested potential asymmetry, and the presence of an influential outlier complicates interpretation.

### Implications for Practice and Policy

The study findings carry clear implications for clinicians, public health officials, and policymakers in GCC countries. First, the high prevalence and incomplete awareness data point to an urgent need for structured screening programs, particularly targeting reproductive-aged women attending primary care settings, universities, and workplace health programs. Second, given the strong association between obesity and PMOS, preventive strategies addressing diet, physical activity, and metabolic health should be integrated into existing non-communicable disease frameworks. Third, our meta-regression findings reinforce the call for regional standardization of PMOS diagnostic criteria. Without this, comparing studies, tracking trends over time, or evaluating interventions will remain impossible. The Rotterdam criteria, despite their complexity, have the broadest international validation and offer the most clinically comprehensive framework [61]. Finally, the mental health dimension of PMOS should not be overlooked. Women with PMOS face significantly elevated rates of anxiety and depression, yet only a handful of included studies even mentioned psychological well-being. Integrating mental health screening into PMOS care pathways would represent a meaningful step forward for the region.

## 6. Conclusions

This systematic review and meta-analysis suggest that approximately one in six women in GCC countries may be living with PMOS. The wide variation in reported prevalence across studies is largely driven by inconsistent diagnostic criteria, heterogeneous study settings, and the reliance on self-report diagnosis in many studies. Until GCC countries adopt standardized diagnostic protocols and invest in population-level epidemiological data, the true scale of the problem will remain obscured, and public health responses will remain inadequate. The clinical picture revealed a high rate of menstrual irregularity, hirsutism, acne, and obesity, compounded by limited awareness of the condition. Strengthening health literacy around PMOS, expanding access to multidisciplinary care, and embedding PMOS screening within existing primary care and reproductive health services are practical and achievable priorities. Future research in the GCC should move beyond cross-sectional prevalence studies toward longitudinal cohort designs that can track disease trajectories, metabolic complications, and treatment outcomes over time. Studies should prioritize representative sampling, validated diagnostic tools, and the inclusion of understudied populations including older women, rural communities, and non-national residents to build a more complete and actionable evidence base.

## Figures and Tables

**Table 1:** Search strategy for literature review on polyendocrine metabolic ovarian syndrome prevalence in Gulf Countries.

Concept	Search Terms
Condition (PCOS)	(“PCOS” OR “Polycystic Ovary Syndrome” OR “Polycystic Ovarian Syndrome” OR “Sclerocystic Ovarian Degeneration” OR “Sclerocystic Ovary Syndrome” OR “Stein-Leventhal Syndrome” OR “Stein Leventhal Syndrome” OR “Sclerocystic Ovaries” OR “Sclerocystic Ovary”)
Epidemiological Outcomes	(“Prevalence” OR “Epidemiology” OR “Frequency” OR “Rate” OR “Burden”)
Geographic Region	(“Saudi Arabia” OR “Saudi” OR “United Arab Emirates” OR “UAE” OR “Emirati” OR “Oman” OR “Omani” OR “Kuwait” OR “Kuwaiti” OR “Qatar” OR “Qatari” OR “Bahrain” OR “Bahraini”)

**Table 6:** Model Comparison Summary.

Model	QM	df	*p*-Value	AIC	BIC	I^2^ (%)	R^2^ (%)
Null Model	—	—	—	64.98	67.25	98.8	—
Assessment Tool	21.01	5	0.0008	52.44	58.67	97.0	42.7
Country	7.85	4	0.097	59.35	65.02	98.2	14.2
Setting	11.35	3	0.010	56.93	61.91	98.1	27.8
Assessment + Country	30.16	9	0.0004	49.99	57.02	95.8	49.2

QM = Test of moderators (omnibus test); df = Degrees of freedom; AIC = Akaike Information Criterion; BIC = Bayesian Information Criterion; I^2^ = Percentage of total variability attributable to between-study heterogeneity rather than sampling error; R^2^ = Proportion of between-study variance explained by the moderator(s); Null model = Random-effects model without moderators. Lower AIC and BIC values indicate better model fit. Significant QM values (*p* < 0.05) indicate that the moderator significantly explains variation in prevalence estimates. R^2^ was calculated relative to the null model and represents the percentage reduction in between-study variance (τ^2^). I^2^ values > 75% indicate considerable heterogeneity.

**Table 7:** Results from Separate Univariable Meta-Regression Models for Each Moderator (Assessment Tool, Setting, Country) and the Combined Multivariable Model (Assessment Tool + Country).

Moderator	Category	β (95% CI)	SE	*p*-Value
Assessment Tool	Rotterdam/NIH (Reference)	Reference	—	—
	Self-report	0.46 (−0.22 to 1.13)	0.35	0.187
	Physician diagnosis	0.61 (−0.33 to 1.55)	0.48	0.203
	Ultrasound/Clinical	1.28 (0.18 to 2.38)	0.56	0.022
	ICD-10	−2.30 (−3.73 to −0.87)	0.73	0.002
Setting	Community (Reference)	Reference	—	—
	Academic	−0.54 (−1.24 to 0.16)	0.36	0.133
	Clinical	−1.27 (−2.18 to −0.37)	0.46	0.006
	Other	1.02 (−0.57 to 2.61)	0.81	0.209
Country	Kuwait (Reference)	Reference	—	—
	Oman	−1.16 (−3.20 to 0.88)	1.04	0.265
	Qatar	−0.11 (−2.13 to 1.91)	1.03	0.913
	Saudi Arabia	0.42 (−1.27 to 2.12)	0.87	0.626
	UAE	−0.39 (−2.22 to 1.45)	0.94	0.68
Combined Model (Assessment Tool + Country)	Intercept	−1.95 (−3.68 to −0.22)	0.88	0.027
	Self-report	−0.10 (−0.96 to 0.77)	0.44	0.827
	Physician diagnosis	0.31 (−0.86 to 1.48)	0.6	0.602
	Ultrasound/Clinical	0.71 (−0.49 to 1.92)	0.62	0.247
	ICD-10	−2.75 (−4.32 to −1.18)	0.8	0.001
	Oman	−0.84 (−2.81 to 1.14)	1.01	0.407
	Qatar	0.20 (−1.76 to 2.15)	1	0.845
	Saudi Arabia	0.69 (−0.93 to 2.31)	0.83	0.405
	UAE	0.57 (−1.05 to 2.19)	0.83	0.49

Note: Estimates (β) are on the logit scale. Positive values indicate higher prevalence compared to the reference category; negative values indicate lower prevalence.

## Data Availability

The original contributions presented in this study are included in the article. Further inquiries can be directed to the corresponding author.

## Abbreviations

Polycystic ovary syndrome (PCOS), Gulf Cooperation Council (GCC), Disability-Adjusted Life Years (DALYs), Preferred Reporting Items for Systematic Reviews and Meta-Analyses (PRISMA).
